# Interactive whiteboard use in clinical reasoning sessions to teach diagnostic test ordering and interpretation to undergraduate medical students

**DOI:** 10.1186/s12909-019-1834-1

**Published:** 2019-11-15

**Authors:** Fares Gouzi, Christophe Hédon, Léo Blervaque, Emilie Passerieux, Nils Kuster, Thierry Pujol, Jacques Mercier, Maurice Hayot

**Affiliations:** 1 PhyMedExp, INSERM U1046, CNRS UMR 9214, Montpellier University HospitalMontpellier University, F-34295 Montpellier, France; 2 French College of University Teachers in Health, (College Français des Enseignants Universitaires de Physiologie en Santé – CFEUPS), Montpellier, France; 3 Laboratoire d’Innovation Pédagogique et de Création d’Outils Multimédia (LIPCOM), Montpellier University, Montpellier, France

**Keywords:** Laboratory radiology test, Clinical reasoning, Technology, Cognitive load

## Abstract

**Background:**

Over-testing of patients is a significant problem in clinical medicine that can be tackled by education. Clinical reasoning learning (CRL) is a potentially relevant method for teaching test ordering and interpretation. The feasibility might be improved by using an interactive whiteboard (IWB) during the CRL sessions to enhance student perceptions and behaviours around diagnostic tests. Overall, IWB/CRL could improve their skills.

**Methods:**

Third-year undergraduate medical students enrolled in a vertically integrated curriculum were randomized into two groups before clinical placement in either a respiratory disease or respiratory physiology unit: IWB-based CRL plus clinical mentoring (IWB/CRL + CM: *n* = 40) or clinical mentoring only (CM-only: *n* = 40). Feasibility and learning outcomes were assessed. In addition, feedback via questionnaire of the IWB students and their classmates (*n* = 233) was compared.

**Results:**

Analyses of the IWB/CRL sessions (*n* = 40, 27 paperboards) revealed that they met validated learning objectives. Students perceived IWB as useful and easy to use. After the IWB/CRL + CM sessions, students mentioned more hypothesis-based indications in a test ordering file (*p* <  0.001) and looked for more nonclinical signs directly on raw data tests (*p* <  0.01) compared with students in the CM-only group. Last, among students who attended pre- and post-assessments (*n* = 23), the number of diagnostic tests ordered did not change in the IWB/CRL + CM group (+ 7%; p = N.S), whereas it increased among CM-only students (+ 30%; *p* <  0.001). Test interpretability increased significantly in the IWB/CRL + CM group (from 4.7 to 37.2%; *p* <  0.01) but not significantly in the CM-only group (from 2.4 to 9.8%; *p* = 0.36).

**Conclusions:**

Integrating IWB into CRL sessions is feasible to teach test ordering and interpretation to undergraduate students. Moreover, student feedback and prospective assessment suggested a positive impact of IWB/CRL sessions on students’ learning.

## Background

Laboratory, radiology, functional and nuclear medicine tests are affordable tools in modern medicine [1]. However, concern about the overuse of these techniques has been growing [2–4]. For instance, the contribution of laboratory investigations to final diagnosis remains lower than medical history and clinical examination [5]. In addition, recent retrospective reports have shown the high prevalence of inappropriate and avoidable tests [3, 6–9]. Over-ordering increases patient discomfort and harm related to iatrogenesis [7, 9], may result in false-positive results, and wastes healthcare resources [3].

Inappropriate test ordering can be improved by education and training in test requests, interpretation and use [3, 4], as the inappropriateness is often due to the physician’s uncertainty about the test indications, performance, feasibility, contraindications, and risk, as well as a lack of knowledge about better alternatives [10]. In addition, education in clinical assessment (pre-analytical) and interpretation of first-line diagnostic tests may reduce the need for more invasive and expensive tools.

Studies have shown that long-term education programs can improve future clinical practice [11]. Thus, early instruction in test ordering and interpretation for undergraduate medical students in embedded courses has emerged as a relevant educational strategy [3]. Harendza et al. showed that students in a vertically integrated curriculum (having learned to identify the clinical question, the technical and diagnostic performance of tests, and how test results impact decisions) [12] ordered fewer diagnostic tests than those in a traditional curriculum [13]. In line with this strategy, clinical reasoning learning (CRL) might help build skills in test ordering and interpretation. CRL is an extension of the problem-based learning approach for medical and clinical problems [14–16]. It encourages students to mobilize and reorganize their knowledge [17] and provides remediation for students with clinical reasoning difficulties [18]. With this method, clinical assessment and test ordering and interpretation are embedded in small group training. In the most prevalent CRL approach (serial-cue), one student simulates a previously examined patient and progressively reveals the clinical signs or diagnostic tests to the other students. In a recent study, CRL improved student perceptions of their ability to request relevant and hypothesis-based tests [19]. However, training in test interpretation has never been evaluated in CRL sessions.

Interactive whiteboards (IWBs) might improve the integration of diagnostic test training into CRL sessions and further improve learning about test ordering, interpretation and use by undergraduate medical students. IWBs provide access to digital material like diagnostic tests and have the following benefits: they can be annotated (as shown for imaging [20]), they enable the scaffolding of decision trees [21], and they promote collaborative learning. Although IWBs were shown to be feasible for problem-based learning in health sciences education [22], their feasibility in CRL sessions has never been tested. The aims of our study were thus:

1. To assess the feasibility of IWB/CRL sessions to teach test ordering and interpretation to third-year undergraduate medical students.
2. To compare the feedback from these medical students with the feedback from third- to sixth-year medical students who followed traditional courses on test ordering and interpretation.
3. To compare improvements in the appropriateness of test ordering and interpretation in third-year undergraduate medical students after 2 months of a vertically integrated module with and without IWB/CRL sessions.

## Methods

### Participants and educational context

IWB/CRL sessions were delivered to third-year undergraduate medical students at the Montpellier-Nîmes School of Medicine, Montpellier University. In the French system, students enter medical school directly after high school to begin medical studies. There is thus no “pre-medical” program. The curriculum was vertically integrated and grouped into several modules. During the 2-month module on cardiovascular and respiratory disease, students attended a morning clinical placement in the respiratory disease or respiratory physiology unit of Montpellier University Hospital for 7 weeks. During the placement, five 1-h courses on test ordering and interpretation were delivered. In addition, all students received individual clinical mentoring by senior physicians that included professional development, support, and guidance in pursuing research or specialization [23]). Students also attended theoretical classroom courses and supervised work sessions every afternoon at the medical school. The French College of University Teachers in Health has validated a reference course on diagnostic test ordering and interpretation for medical students (http://side-sante.org/sites/default/files/Prescription%20Explorations%20Fonctionnelles%20Physiologiques.pdf). In line with this course and other publications, the objective of equipping medical students to request, interpret and utilize diagnostic tests is to ensure that they are able to:

1. Clinically assess the patient.
2. Identify the question/the indication.
3. Suggest one or more hypotheses.
4. Assess potential contraindications, feasibility, requirements and risks of the diagnostic test.
5. Discuss potential alternatives to the diagnostic test.
6. Specify the conditions for carrying out the test.
7. Inform the patient.
8. Verify the interpretability of the results.
9. Compare the results with the reference values, previous personal values, and post-challenge values.
10. Interpret the results: use the positive and negative results/paraclinical signs to answer the question − affirm or eliminate the hypothesis.

### Study design

#### Study 1 (part 1): feasibility

Third-year undergraduate medical students at the Montpellier-Nîmes School of Medicine enrolled in the 2-month module on cardiovascular and respiratory medicine were randomly assigned to a respiratory disease or a respiratory physiology unit. Randomization was performed in blocks of 20 students. A computer-generated list of random numbers was used to assign the students to the study groups. Each student assigned to the respiratory physiology unit received four IWB/CRL sessions (90 min each) and clinical mentoring (IWB/CRL + CM group), while students assigned to the respiratory disease unit followed the traditional curriculum with clinical mentoring only (CM-only group). The feasibility of the IWB/CRL sessions was assessed by analysing the paperboards from the sessions.

#### Study 2: student feedback

Through their university email addresses, all medical students at the Montpellier-Nîmes School of Medicine were invited to answer an online questionnaire (Additional file 1. Questionnaire). The questionnaire responses were compared with those of the third-year medical students after their last session of IWB/CRL.

#### Study 1 (part 2): learning outcome assessment

During placements in the respiratory disease and respiratory physiology units, students learned about the diagnostic tests used in the field of respiratory medicine (spirometry, exercise testing, sleep tests, imaging, endoscopy). Students in both groups were asked to clinically examine their patients and report the findings individually. The respiratory disease and respiratory physiology units shared the same patients. Learning was assessed by prospectively comparing the appropriateness of test ordering and interpretation in both student groups with a 1-h test at the beginning and end of their 2-month clinical placement.

### IWB/CRL sessions

The CRL sessions were run in small groups of six to eight students, with a physician or resident as the facilitator [16]. A real clinical encounter was simulated using the serial-cue method. While the students “played the doctor” and actively gathered information necessary for diagnosis, one student “played the patient” and progressively revealed clinical signs. The students recorded the information on the IWB. The facilitator regularly asked them why they had requested certain clinical information or ordered a test to prompt them to express/write their clinical questions and hypotheses on the IWB. The facilitator was also able to gain access to their reasoning and correct errors by asking them how the clinical or other information would help to affirm/reject a diagnostic hypothesis. The diagnostic tests that were ordered were written on the IWB and discussed. Then, the raw test data (previously digitized in JPEG) were provided onscreen and any signs underlying the clinical manifestations were annotated for further interpretation. Last, the facilitator asked the students to return to the simulated patient’s initial complaint and the clinical questions/hypotheses so that they could prepare a summary and conclude the IWB/CRL session. The final paperboard of the session was then provided to the students as a .pdf file.

### Analysis of the IWB/CRL sessions (study 1; part 1)

A review team of three teachers (FG + CH + MH) predefined items that matched the validated course objectives for diagnostic test ordering and interpretation as set by the French College of University Teachers in Health. The .pdf files of the paperboards from the IWB/CRL sessions were then blindly reviewed by two teachers (FG + CH), and all items were assessed. Last, the agreement of the two teachers’ assessments was rated for each item. Depending on the item, inter-teacher agreement was fairly good to excellent, with Lin concordance coefficients from 0.86 [0.68–1.04] to 0.97 [0.91–1.01].

### Student feedback on test ordering and interpretation courses (study 2)

At the end of the clinical placement, students who participated in the IWB/CRL sessions were asked to respond to an online questionnaire (10 min; Additional file 1. Questionnaire). The questions were designed to determine whether the students had indeed learned test ordering and interpretation as set out in the course objectives of the French College of University Teachers in Health. In addition, four questions about perceptions of the IWB were added, in accordance with the technology acceptance model [24]. Perceived usefulness (utility) and perceived ease of use (usability) were assessed with Likert scales for each dimension [25]. Questions also addressed student perceptions of their curriculum, practice and self-confidence in diagnostic test ordering and interpretation. In parallel, this online questionnaire was administered to all third- through sixth-year students following the vertically integrated curriculum.

### Examination and assessment of the learning outcomes (study 1; part 2)

At the beginning and end of their clinical placement, the third-year medical students were invited to attend a 1-h group assessment session (optional). In the session, four clinical cases of patients with real-life respiratory disease were presented in the same format as during the IWB/CRL sessions. Again, as set out in the course objectives of the French College of University Teachers in Health, the clinical question was clearly specified (3 diagnoses and 1 follow-up) and the students were asked to write their hypotheses and then diagnostic tests (specifying the indications, risks, limits and modalities) in a standardized form. Each case presentation was thus followed by two or three diagnostic tests, and the students had to write their test interpretation, specifying positive and negative signs (if possible). The four cases were the same (in terms of content) in the pre- and post-test assessments. The assessment session was supervised by a teacher. On each student’s form, the correspondence between the diagnostic tests and the hypotheses, and the classic test indications, were reviewed. The correspondence between tests and hypotheses was defined as:

- The number of ordered tests that could affirm or eliminate a hypothesis;
- The ratio between ordered tests that could affirm or eliminate a hypothesis and all tests ordered. The correspondence between tests and indications was defined as:
- The number of ordered tests that were validated for an indication, as defined in the French reference document on respiratory diseases for undergraduate students (http://cep.splf.fr/enseignement-du-deuxieme-cycle-dcem/referentiel-national-de-pneumologie/);
- The ratio between tests validated for an indication and all tests ordered.

Test identification and interpretability were recorded. Last, the number of signs underlying the clinical manifestations and the appropriateness of the hypotheses were also recorded. The answers were blindly reviewed by the two teachers. Depending on the item, the inter-teacher agreement was fairly good to excellent, with Lin concordance coefficients from 0.98 [0.97–0.99] to 0.88 [0.82–0.90].

### Statistical analysis

The anonymized data were statistically analysed. Quantitative data were presented as means ± SD or medians [IQR 25–75] depending on the results of the Kolmogorov-Smirnov test of normality. For the feasibility and feedback studies, quantitative data were analysed using a one-way ANOVA and an independent *t*-test. Qualitative data were analysed with the Kruskal-Wallis test, a two-proportion Z test and Fisher’s exact test. For multiple comparisons, a Bonferroni correction was performed. In the learning outcome study, two groups (IWB/CRL + CM and CM-only) were assessed twice (before and after 7 weeks of clinical placement), with four clinical cases. Thus, data were analysed with a multilevel linear mixed effect model, with two nested levels of random effects, the student identity (Level 1) and the clinical case (Level 2), to take into account the dependency of the data [1]. In this model, we used the Time (T) and Group (G) effects, as well as the interaction between these factors (GxT), as fixed effects. The analyses were completed with Fisher’s LSD post-hoc test when the Group x Time interaction term was significant. The normal distribution of the residual was verified with a Q-Q plot for each model. Data were analysed with R 3.5.0 software (www.r-project.org). A *p*-value < 0.05 was considered significant.

## Results

### Feasibility of the IWB/CRL sessions

**Fig. 1:**
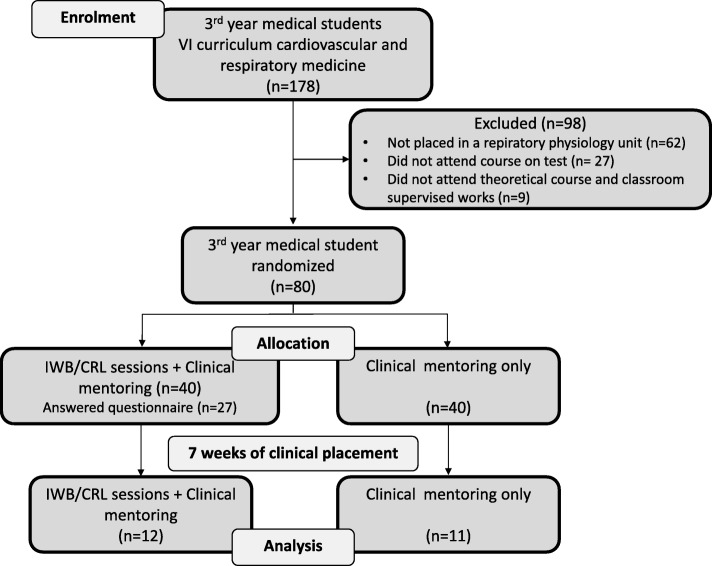
Flowchart of Study 1: Feasibility and learning performance assessment

**Fig. 2:**
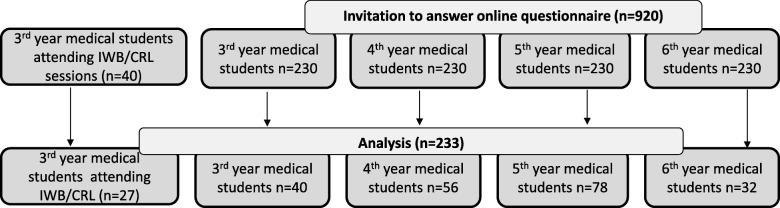
Flowchart of Study 2: student’s feedback

From September 2016 to October 2018, 178 out of the 230 third-year students who had been screened (127 women and 103 men) were eligible; all were in the vertically integrated curriculum and enrolled in a cardiovascular and respiratory disease module, as depicted in Fig. 1. Forty of these 178 (26 women and 14 men) participated in 22 IWB/CRL sessions (mean number: 3.78 ± 1.09). The day after the end of the module and clinical placement, 27 of these 40 students (13 women and 14 men) responded to the feedback study questionnaire. Essentially, 40 out of the 178 eligible students were randomly assigned to the IWB/CRL + CM group and 40 to the CM-only group. All 80 participated in the pre-training assessment, but only 23 (13 women and 10 men) participated in the post-training assessment. Gender (F/M) did not significantly differ for eligible students (55%/45%), students in the IWB/CRL + CM group (65%/35%; *p* = 0.291), students who responded to the questionnaire (48%/52%; *p* = 0.539), or students who participated in pre- and post-tests (56%/44%, *p* = 1.00). The attrition rate was not significantly different between groups (70% vs. 72.5%, p = 1.00) and the characteristics of the students who dropped out and those who completed the post-test assessment did not differ in terms of gender or prior knowledge in respiratory basics (Additional file 2: Table S1.). The questionnaire responses of those who attended the IWB/CRL sessions were compared with the responses of the other third- through sixth-year medical students (Fig. 2). Two hundred and thirty-three students representing 22% of all medical students at the Montpellier-Nîmes School of Medicine responded to the questionnaire.

**Fig. 3:**
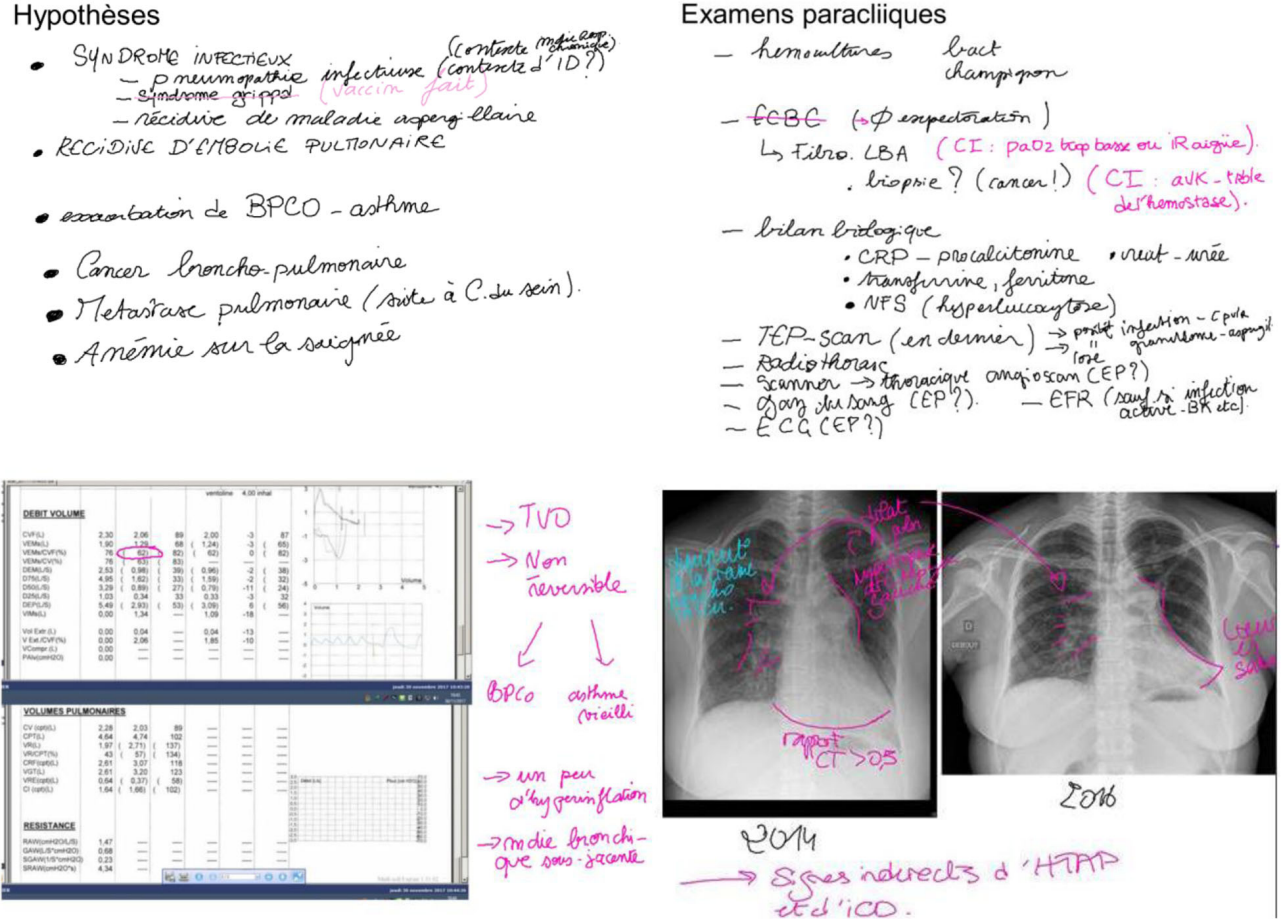
Screenshots of the paperboards from the IWB-based CRL sessions. Hypothese: hypothesis; syndrome infectieux: infectious syndrome; récidive d’embolie pulmonaire: pulmonary embolism recurrence; exacerbation de BPCO-asthme: acute exacerbation of chronic obstructive pulmonary disease or asthma; cancer bronchopulmonaire: bronco pulmonary cancer; metastase pulmonaire: pulmonary metastasis; anémie sur la saignée = bloodletting-induced anaemia; examens paracliniques: diagnostic tests; hemocultures: blood cultures; ECBC: sputum analysis; fibro-LBA-biopsie: bronchoscopy-bronchiolo-alveolar lavage-biopsy; bilan biologique: laboratory blood tests

The number of students per IWB/CRL session was between four and seven, and sessions lasted between 60 and 90 min each. One student (3.7%) never played the doctor’s role, six (22%) played the doctor’s role, and 20 (74%) played both roles (Fig. 3).

The .pdf files of the IWB/CRL sessions revealed the questions, hypotheses, and diagnostic tests suggested by the students, as well as the extra-clinical signs identified on the raw test data. The quantitative analysis based on the learning objectives set by the French College of University Teachers is presented in Table 1. As expected, clinical questions and hypotheses were systematically recorded on the IWB. During sessions, 4.36 ± 1.59 diagnostic tests of all types were presented on the IWB. While interpretability was not systematically discussed, 8.57 ± 5.27 extra-clinical signs were identified and generally used to answer questions. The questionnaires revealed that most students responded that they “agreed” or “strongly agreed” about IWB usefulness and ease of use (Table 2; 96, 70, 81, 85%, for questions 1 to 4, respectively).

**Table 1:** Analysis of the IWB/CRL sessions

Objectives of learning test requesting, interpretation and use for medical students	*N* = 22
1. Clinically assess the patient	100%
2. Identify the question/the indication	100%
No. of questions/indications	2.48 ± 1.34
Type % (diagnosis/aetiology/prognosis/evolution/complication/treatment)	35/70/35/22/65/9
Suggestion of a diagnostic test	100%
No. of diagnostic tests suggested	8.05 ± 3.21
Mention of an indication/test	63.6%
*• Test a hypothesis*	90.9%
*• Conform to recommendations*	0.0%
*• “Systematic approach”*	0.0%
*• Assess the time course*	63.6%
*• Adapt the treatment*	4.5%
Appropriateness of the test regarding the diagnostic hypothesis	
Mention of the looked-for nonclinical signs/test	26.1%
3. Suggest one or more hypotheses	100%
No. of hypotheses	7.23 ± 2.27
4. Assess the potential contraindications, feasibility, requirements and risks of the diagnostic test	
Risks and adverse events discussed	30.4%
Feasibility and limits discussed	17.4%
6. Specify the conditions for conducting the test	31.3%
No. of diagnostic tests interpreted	4.36 ± 1.59
*• Biology/laboratory*	0.90 ± 0.89
*• Functional*	2.09 ± 1.27
*• Imaging*	1.09 ± 0.68
*• Nuclear medicine*	0.05 ± 0.21
*• Cytology – Anatomic pathology*	0.05 ± 0.21
*• Other*	0.05 ± 0.21
8. Verify the interpretability of the results	23.8%
9. Compare the results with reference values, previous personal values, post-challenge values	100%
No. of nonclinical signs identified	8.57 ± 5.27
*• No. of positive signs identified*	5.48 ± 3.14
*• No. of negative signs identified*	3.10 ± 2.93
10. Interpret the results: use signs to answer the question – affirm or eliminate the hypothesis	54.5%

**Table 2:** IWB usefulness and ease of use

	IWB/CRL *N* = 27
1. Was the IWB easy to use?	4.37 ± 0.56
2. Was the IWB useful to learn diagnostic test ordering?	3.89 ± 0.89
3. Was the IWB useful to learn diagnostic test interpretation?	4.11 ± 0.80
4. Was the IWB a useful tool to learn how to use the diagnostic test in clinical situations?	4.11 ± 0.64

### Comparison of student feedback: IWB/CRL sessions vs. traditional courses with CM-only

In parallel, 206 students in traditional courses responded to the online questionnaire from September 2017 to January 2018. Bedside learning/mentoring together with classroom instruction and self-study was the most prevalent way of learning about diagnostic tests and their interpretation. Student’s feedbacks are presented in Table 3. The need for more courses or training sessions on diagnostic test ordering and interpretation was expressed by 92.5, 98.2, 95.9, 93.8%, of the students from the third to sixth year, respectively (Additional file 2: Table S2.). Although students could systematically suggest diagnostic tests during the IWB/CRL sessions, students from the third to sixth year reported this as occurring “never” to “systematically” as follows: from 37.5 to 96.9% in clinical examination reports, from 35 to 90.6% at bedside with a resident/senior physician, from 47.5 to 87.5% in clinical case presentations, from 90 to 87.5% during classroom instruction, and from 17.9 to 65.6% during medical staff meetings (Additional file 2: Table S2).

**Table 3:** Student feedback about test ordering and interpretation. IWB/CRL sessions vs. traditional learning sessions

	IWB/CRL sessions	Vertically integrated curriculum				*p*
	3rd year *n* = 27	3rd year *n* = 40	4th year *n* = 56	5th year *n* = 78	6th year *n* = 32	
1. Now when I complete a test ordering file, I understand the reason/indication for the test	3.37 ± 1.01	3.95 ± 0.83	3.68 ± 0.79	4.01 ± 0.81	4.50 ± 0.62*	< 0.001
2. Now when I complete a test ordering file, the most frequent reason/indication that I specify is (%):						
*• I never specify a reason or indication/I specify the resident/senior’s request*	0.0	61.5	57.1	64.1	40.6	< 0.001
*• To test (affirm or eliminate) a hypothesis*	27.9	10.3	17.9	26.9	46.9	
*• To conform to recommendations*	23.0	12.8	21.4	6.4	9.4	
*• As “a systematic approach”*	8.2	5.1	0	2.6	0	
*• To assess the time course*	29.5	5.1	3.6	0	3.1	
*• To adapt the treatment*	11.5	5.1	0	0	0	
3. Now when I complete a test ordering file, I specify one or more nonclinical signs to be looked for	2.81 ± 1.17	2.51 ± 1.10	2.63 ± 1.10	3.14 ± 1.18	3.61 ± 1.12*	< 0.001
4. Now when I complete a test ordering file, I integrate the risks and limitations into the decision	3.46 ± 1.07	NK	3.04 ± 0.85	3.50 ± 0.83#	3.72 ± 0.77#	0.002
5. Now I look for positive and negative nonclinical signs directly on the raw data and not on the report	3.54 ± 1.03**	2.77 ± 0.87	2.48 ± 0.85	2.70 ± 0.92	3.47 ± 0.88**	< 0.001

Student feedback on test ordering and interpretation. IWB/CRL sessions vs. traditional learning sessions. Data are presented as mean ± SD on a Likert scale (1: never; 2: rarely; 3: regularly; 4: often; 5: systematically)

Sixth-year vs. other groups *p<0.05

Sixth-year and fifth-year vs. fourth-year #*p*<0.05

Third-year IWB/CRL+CM group and sixth-year vs. other groups **p<0.05

**Fig. 4:**
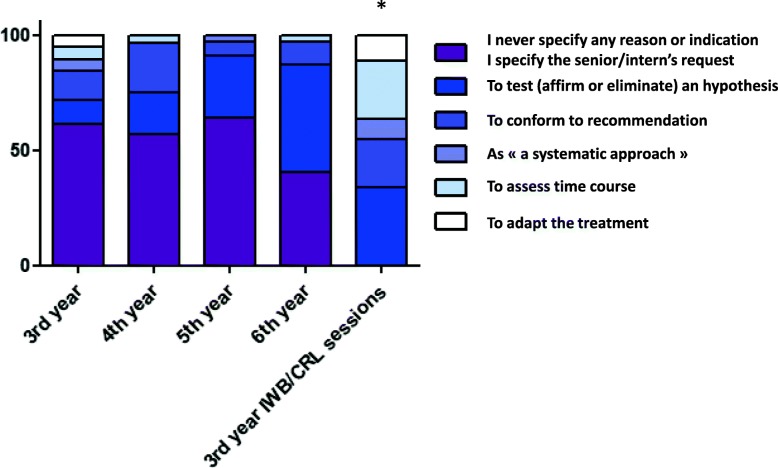
Student feedback about test ordering and interpretation. IWB/CRL sessions vs. traditional learning sessions for the answer to the question: “Now when I complete a test ordering file, the most frequent reason/indication that I specify is: … ”. Data are presented in proportions for each answer (%). Third-year IWB/CRL + CM group vs. other groups; *p* <  0.05

The online questionnaires showed an increase in perceived understanding of the reason/indication for tests over the course of study, with 37.5% of the sixth-year students answering “frequently” and 56.3% of them “systematically” (*p* <  0.05 vs. other groups). The third-year students who participated in the IWB/CRL sessions reported practices similar to those of more advanced students in terms of specifying the extra-clinical signs to look for, considering risks and limitations, and directly analysing the raw test data. Although the answers were not compared to those of other students, the third-year IWB/CRL + CM students declared verifying test interpretability as follows: “regularly” for 40.7%, “often” for 22.2%, and “systematically” for 7.4%. After the IWB/CRL sessions, these students completed diagnostic test ordering files with indications for diagnostic tests that significantly differed from those of their classmates (*p* <  0.01). In particular, 27.9% of them stated it was “to test (affirm or eliminate) a hypothesis,” whereas this was the case for 10.3, 17.9, 26.9 and 46.9% of the third- to sixth-year students, respectively (Fig. 4).

### Learning outcomes in the IWB/CRL sessions vs. traditional sessions with CM-only

**Fig. 5:**
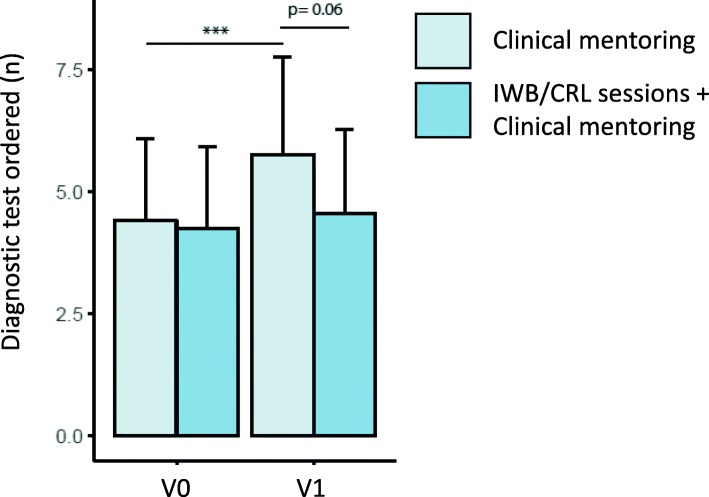
Change in pre- and post-test in the IWB/CRL + CM group and the CM-only group. ***p <  0.001

The prospective study was offered to third-year undergraduate medical students assigned to the respiratory disease and respiratory physiology units of Montpellier University Hospital. Although 80 students attended the pre-assessment tests, only 23 students completed both the pre- and post-tests. A significant time effect was observed for the number of suggested hypotheses; the number of clear indications specified; the mention of test requirements; and the matches between tests and hypotheses, indications and hypotheses, and tests and indications, which pointed to the significant improvement in these parameters for all students over time (Table 4). The number of extra-clinical signs identified also improved for all students. A significant Time*Group interaction was observed for the number of diagnostic tests ordered (*p* <  0.01, Fig. 5), increasing only in the CM-only group with traditional courses [from 4.42 ± 1.67 to 5.76 ± 2.01 (+ 30%); *p* <  0.001], whereas it did not change in the IWB/CRL + CM group (from 4.25 ± 1.67 to 4.55 ± 1.73: + 7%; *p* = N.S). In addition, mentions of test interpretability increased significantly in the IWB/CRL + CM group (from 4.7 to 37.2%; p <  0.01), whereas it did not significantly change in the CM-only group (from 2.4 to 9.8%; *p* = 0.36; Table 5).

**Table 4:** Change in pre- and post-test in IWB/CRL + CM group and CM-only group

	IWB/CRL sessions		Traditional learning sessions		
	*N* = 12		*N* = 11		
	V0	V1	V0	V1	P
Hypothesis proposed (n)	2.33 ± 1.26	2.89 ± 1.36	2.53 ± 1.32	2.98 ± 1.42	T: < 0.001
Diagnostic test ordered (n)	4.25 ± 1.67	4.55 ± 1.73	4.42 ± 1.67	5.76 ± 2.01	G*T: < 0.01
Clear indication specified (n)	1.81 ± 1.81	2.66 ± 2.04	1.58 ± 1.61	2.56 ± 1.91	T: < 0.001
Risk and limits mentioned (n)	0.35 ± 0.70	0.38 ± 0.97	0.71 ± 0.99	0.95 ± 1.43	N.S
Test requirements mentioned (n)	0.17 ± 0.52	0.23 ± 0.70	0.37 ± 0.76	0.68 ± 1.29	T: 0.07
Correspondence between test and hypothesis					
Number of appropriate tests (n)	2.77 ± 1.8	3.47 ± 1.79	2.72 ± 1.55	3.83 ± 2.01	T: < 0.001
Ratio of appropriate tests	1.32 ± 0.92	1.29 ± 0.64	1.21 ± 0.88	1.33 ± 0.64	N.S
Correspondence between indication and hypothesis					
Number of appropriate tests (n)	1.15 ± 1.35	1.98 ± 1.79	0.98 ± 1.12	1.72 ± 1.43	T: < 0.001
Ratio of appropriate tests	0.53 ± 0.61	0.70 ± 0.71	0.45 ± 0.56	0.59 ± 0.44	T: 0.07
Correspondence between test and indication					
Number of appropriate tests (n)	1.63 ± 1.65	2.51 ± 1.9	1.33 ± 1.39	2.21 ± 1.72	T: < 0.001
Ratio of appropriate tests	0.39 ± 0.38	0.52 ± 0.36	0.32 ± 0.31	0.40 ± 0.29	T: < 0.001
Extra-clinical signs found					
Number of true extra-clinical signs (n)	1.63 ± 1.14	2.57 ± 1.72	1.29 ± 1.11	2.05 ± 1.66	T: < 0.001
Number of extra-clinical signs consistent with the proposed hypothesis (n)	0.60 ± 0.76	1.23 ± 1.07	0.52 ± 0.74	1.07 ± 1.33	T: < 0.001

Change in pre- and post-test in the IWB/CRL+CM group and CM-only group. Data are presented as mean ± SD. T: Time effect. G*T: Group-Time effect. N.S: Not significant

**Table 5:** Change in pre- and post-test in IWB/CRL group and CM-only group

	IWB/CRL sessions N = 12			Traditional learning sessions N = 11				
	V0	V1	*P intra*	V0	*P inter*	V1	*P inter*	*P intra*
Identification of the diagnostic test (1/0)	56.5%	80.4%	*0.096*	58.5%	*1.000*	65.9%	*0.592*	*N.S*
Interpretability (1/0)	4.7%	37.2%	*0.004*	2.4%	*1.000*	9.8%	*0.016*	*N.S*

## Discussion

This study shows the feasibility of using an IWB during CRL sessions to teach diagnostic test ordering and interpretation. The sessions were feasible for undergraduate medical students, who met the validated learning objectives. To our knowledge, this is the first study in medical education showing a change in students’ perceived practices and skills in test ordering and interpretation.

### IWB for CRL sessions: potential and feasibility for achieving learning objectives

In previous systematic reviews outside the medical field, adding the IWB to problem-based learning methods was shown to have potentially positive effects on learning [22, 26]. Doing so appears to improve data sharing and the observation of nonclinical signs [20, 21] and it may also scaffold reasoning [27], enquiry learning and hypothesis generation [28]. Our study confirmed this potential in the medical education field. Although we did not compare learning in the IWB/CRL sessions versus the CRL sessions alone, 8.57 ± 5.27 nonclinical signs were observed in 4.36 ± 1.59 tests in IWB/CRL. In addition, hypotheses/tests were systematically suggested, and the observed nonclinical signs were used to affirm or eliminate a hypothesis in more than 50% of the sessions. Thus, the IWB/CRL sessions seemed to enhance student participation, as indicated by the detail on the IWBs and in contrast to the student feedback on the traditional medical curriculum, regardless of the teaching modality, as previously shown [28, 29].

Our interest in assessing the feasibility of using an educational technology (IWB) during CRL sessions was based on ergonomic principles [24] in the educational sciences [25, 30]. The IWB appeared easy to use and useful for third-year undergraduate medical students. Likert-scale grading was in line with the study of Jain et al. with an older device [21]. Given the methodological limitations of open and questionnaire-based assessments, the usefulness of the IWB was also evaluated by assessing the correspondence between the learning outcomes and the validated learning objectives [25]. Because we analysed the paperboards and not the sessions directly, some of the learning outcomes may have been underestimated and thus the correspondence with the learning objectives might have been even better. Last, as noted above, we did not compare the usefulness of the IWB during CRL sessions with that of traditional CRL sessions. Yet, it seems evident that some of the objectives (verify the interpretability, identify nonclinical signs, etc.) could not have been addressed during our CRL sessions without the use of the IWB. Although other technologies (e.g. multimedia projectors, tablets, smartphones, laptops) [16] might also have augmented clinical reasoning skills or learning during CRL, our study provides quantified feasibility data about IWB use during CRL sessions that should encourage the behavioural intention of learners and teachers.

### Students’ self-reported practices for diagnostic test ordering and interpretation

After the IWB/CRL sessions, the students’ questionnaire responses about test requests and interpretation revealed attitudes or beliefs that differed from those of their classmates. The differences in mentioning the indication and the nonclinical signs to look for agreed with the paperboard observations. Interestingly, the self-perceived understanding of the reason/indication for tests was lower in the third-year students with IWB/CRL sessions than in students following the traditional curriculum. Yet, this agreed with the moderate [31] or poor correlation between self-perceived competence and objective assessment in undergraduate medical students [32–34]. Strikingly, after the IWB/CRL sessions, these third-year students no longer gave unspecified indications. For the CM-only students in the traditional courses, unspecified indications (“I never specify the indication” or “I specify the resident/senior physician’s request” and the “systematic approach”) were quite prevalent and depended on the mentor and the educational context. Detsky et al. pointed out the role of the trainee’s identification of his/her mentor’s practice and an educational system that rewards exhaustivity in medical over-testing [35]. Therefore, our educational approach showed a relevant educational effect.

However, despite the feasibility and the positive student feedback, learning objectives may not have been fully achieved. Indeed, cognitivists have shown that educational technologies can place an extraneous cognitive load on working memory that hampers learning [36–38]. This is a major issue for the IWB, as evidence of the impact on learning is lacking in health sciences education [26]. Therefore, using the Study 1 design, we paired the self-reports with a prospective randomized controlled study.

### Objective improvements in learning outcomes during diagnostic test education

In the prospective randomized controlled study, the students overall showed progress in aligning the tests with hypotheses (reasoning) and indications (knowledge). While CM-students increased the number of diagnostic tests, the students who attended the IWB/CRL sessions did not, despite similar improvement in test appropriateness, indicating that these students were being more thoughtful in their test ordering.

The learning effect might have been directly associated with the use of the IWB during the CRL sessions. Indeed, the paperboard analysis revealed the hypotheses that needed to be affirmed/eliminated and the nonclinical signs that came to the students’ attention. The paperboards were consistent with the student feedback: the students specified more accurate indications and nonclinical signs. Moreover, this greater specificity was in line with studies showing that diagnostic uncertainty [6, 39, 40] and irrational ordering (i.e. not hypothesis-based) are associated with the overuse of diagnostic tests [41, 42]. Conversely, including probabilistic reasoning in education reduces test ordering [43]. The reduction in diagnostic test ordering has been shown in students following a vertically integrated curriculum [13], and our results with third-year students following this type of curriculum demonstrated that it is possible to further improve the accuracy of their test ordering. Nonetheless, it remains to be demonstrated whether this result can be translated into their future medical practice, as with other effective educational strategies [44, 45].

### Perspective: determinants of improvement during IWB-based CRL sessions

The educational background of the medical students was an issue for those teaching diagnostic test ordering/interpretation from the third to the sixth year, as it is for education researchers who study clinical (and nonclinical) reasoning learning [17, 18]. In our study, the students’ experience (age or years of training) did not impact the results of the prospective study. It did, however, reveal a clear shift in perceptions of test ordering and interpretation skills during undergraduate medical study. This raises questions about the timing of our IWB/CRL sessions on diagnostic tests. Because of low class attendance, third-year medical students in France often lack a solid grounding in physiology and semiology, which might limit the benefits of these sessions for these young medical students. In addition, between the first year and the second and third years, there is a shift in the learning paradigm from knowledge-building to the development of reasoning skills. Therefore, students starting the third year may have memorized considerable medical knowledge but not yet acquired the conceptual understanding that would have enabled them to reason about diagnostic testing. Yet, Allen et al. showed that first-year medical students in the North American system were already able to display clinical reasoning during physical examinations [46], and our IWB/CRL sessions on diagnostic tests were probably offered at a time when these students were starting to acquire clinical reasoning skills. In addition, the student expertise level has been shown to impact the cognitive load during learning tasks. Indeed, using single/multiple, redundant/complementary, transitory/fixed, unimodal/multimedia, and interactive/isolated materials in specified/unspecified ways for solving complex/simple problems all require different perceptual and cognitive resources for working memory [36]. Strikingly, the effects are reversed in expert learners. Assessment of the cognitive load through questionnaires or physiological methods would help to better understand these phenomena and adapt IWB/CRL session content to the student level [47].

### Study limitations

This study had several limitations that should be noted. First, the sample size was small, with 40 students in the IWB/CRL + CM group. Nevertheless, this sample was almost half (44.5%) of the eligible students in the module on cardiovascular and respiratory disease. In addition, all students must follow this module in the third undergraduate year of medical study. Thus, to some extent, the study sample represented the entire population of third-year undergraduate medical students, as suggested by the non-significant difference in gender – an indicator of randomization − between all third-year students and those included in Studies 1 and 2. Second, the questionnaire response rate was 67% for the third-year students attending the IWB/CRL sessions and only 22% for other students from the third to sixth year, and this differential response rate may have affected the validity of our findings. While the response rates in academic studies are 55.6+/− 19.7% [48], the response bias still remains an issue in research studies including questionnaires [49]. Third, the attrition rate was high in the randomized study, with only 29% of the students attending the post-test assessment. However, the students who dropped out did not differ from completers for gender or prior knowledge in respiratory basics (physiology, anatomy, histology, semiology, etc.). More importantly, the attrition rate was not differential, which suggests that the dropouts may not have been linked to the intervention but more likely to the lack of reminders sent to the students by the faculty. Nonetheless, this high attrition rate − although not differential − may have altered the study’s external validity. Indeed, those who completed the study may have represented a subgroup of motivated students. This limitation could have been overcome if the faculty had tied the final validation of the clinical placements to participation in these tests. Last, teacher- and unit-dependency (students in the IWB/CRL + CM group assigned to the respiratory physiology unit may have been more aware of diagnostic test issues) might have had an impact. Altogether, the learning effect observed in our pilot study must be confirmed in a larger scale study, involving more teachers in various hospital units.

## Conclusion

Our study demonstrated the feasibility of integrating the IWB into CRL sessions to teach test ordering and interpretation to undergraduate students. The students were able to focus on the learning objectives via the appropriate use of an educational technology and a validated methodology. Moreover, the feedback from these students revealed different medical attitudes and beliefs regarding test ordering and interpretation, indicating that the teaching “messages” had been heeded. Last, although the additional value of the IWB in the CRL sessions was not tested versus CRL alone, these IWB/CRL sessions impacted the students’ test ordering behaviour and interpretation and may indicate a positive effect of this combined strategy on learning.

## Supplementary information

**Additional file 1.** Questionnaire about the clinical reasoning sessions and diagnostic test learning.

**Additional file 2 Table S1.** Characteristics of students who dropped out and who completed Study 1; part 2: Learning outcome assessment **Table S2.** Student feedback about test ordering and interpretation in traditional learning sessions.

## Data Availability

The datasets used and/or analysed during the current study are available from the corresponding author on reasonable request.

## Abbreviations

CRL: Clinical reasoning learning
IWB: Interactive whiteboard
