# Supplementary material for: Interactive whiteboard use in clinical reasoning sessions to teach diagnostic test ordering and interpretation to undergraduate medical students
Source: BMC Med Educ. 2019 Nov 15;19:424. doi: 10.1186/s12909-019-1834-1 (PMC6858719; doi:10.1186/s12909-019-1834-1)
Supplement: Supplementary file 1 — Additional file 1. Questionnaire about the clinical reasoning sessions and diagnostic test learning. [file 12909_2019_1834_MOESM1_ESM.docx]

**Questionnaire about the clinical reasoning sessions and diagnostic test learning**

A clinical reasoning session is a medical learning exercise in which a few students play roles and try to resolve a clinical problem, with a medical teacher who moderates the session. If you have already participated in a clinical reasoning session, you will have to answer these 19 questions (10 minutes). If you cannot answer a question, go on to the next one. Try to be as sincere as possible in your answers.

Thank you very much!

Question 1. What year of study are you in?

- 3^rd^ year
- 4^th^ year
- 5^th^ year
- 6^th^ year

Question 2. Are you…

- A man
- A woman

Question 3. Have you already written up a clinical examination report? Yes / No

Question 4. Have you already suggested a diagnostic test? Yes / No

Question 5. Have you already suggested a hypothesis? Yes / No

Question 6. Have you ever participated in a clinical reasoning session? Yes / No

Question 7. Which hospital unit were you assigned to? /_____________________/

Question 8. Which role did you play?

- Doctor
- Patient
- Both

Question 9. Was the IWB easy to use?

- Strongly disagree
- Disagree
- Neither agree nor disagree
- Agree
- Strongly agree

Question 10. Was the IWB useful to learn diagnostic test ordering?

- Strongly disagree
- Disagree
- Neither agree nor disagree
- Agree
- Strongly agree

Question 11. Was the IWB useful to learn diagnostic test interpretation?

- Strongly disagree
- Disagree
- Neither agree nor disagree
- Agree
- Strongly agree

Question 12. Was the IWB a useful tool to learn how to use the diagnostic test in clinical situations?

- Strongly disagree
- Disagree
- Neither agree nor disagree
- Agree
- Strongly agree

Question 13. Now when I complete a test ordering file, I understand the reason/indication for the test

- Never
- Rarely
- Sometimes
- Regularly
- Systematically

Question 14. Now when I complete a test ordering file, the most frequent reason / indication that I specify is (choose one or more items):

- I never specify a reason or indication
- I specify the intern/senior’s request
- To test (affirm or eliminate) a hypothesis
- To conform to recommendations
- As “a systematic approach”
- To assess the time course
- To adapt the treatment

Question 15. Now when I complete a test ordering file, I specify one or more extra-clinical signs to be looked for

- Never
- Rarely
- Sometimes
- Regularly
- Systematically

Question 16. Now when I complete a test ordering file, I integrate the risks and limitations into the decision

- Never
- Rarely
- Sometimes
- Regularly
- Systematically

Question 17. Now I look for positive and negative extra-clinical signs directly on the raw data and not on the report:

- Never
- Rarely
- Sometimes
- Regularly
- Systematically

Question 18. I verify the interpretability of diagnostic tests:

- Never
- Rarely
- Sometimes
- Regularly
- Systematically

Question 19. I need more courses or training sessions on diagnostic test ordering and interpretation : Yes / No

**Assessment questionnaire for clinical cases testing the students’ diagnosis test learning**

How many hypotheses were proposed (n)? \_____\

How many diagnostic tests were ordered (n)? \_____\

How many clear indications were specified (n)? \_____\

How many risk and limits were mentioned (n)? \_____\

How many test requirements were mentioned (n)? \_____\

Correspondence between test and hypothesis: number of appropriate tests (n)? \_____\

Correspondence between indication and hypothesis: number of appropriate tests (n)? \_____\

Correspondence between test and indication: number of appropriate tests (n)? \_____\

Regarding the extra-clinical signs found:

-number of true extra-clinical signs (n)? \_____\

-number of extra-clinical signs consistent with the proposed hypothesis(n)? \_____\

Was the diagnostic test identified? yes / no

Is there a mention of the test interpretability? yes / no
