# Supplementary material for: Interactive whiteboard use in clinical reasoning sessions to teach diagnostic test ordering and interpretation to undergraduate medical students
Source: BMC Med Educ. 2019 Nov 15;19:424. doi: 10.1186/s12909-019-1834-1 (PMC6858719; doi:10.1186/s12909-019-1834-1)
Supplement: Supplementary file 2 — Additional file 2 Table S1. Characteristics of students who dropped out and who completed Study 1; part 2: Learning outcome assessment Table S2. Student feedback about test ordering and interpretation in traditional learning sessions. [file 12909_2019_1834_MOESM2_ESM.docx]

**Supplementary Table 1. Characteristics of students who dropped out and who completed Study 1; part 2: Learning outcome assessment**

|  | **IWB/CRL sessions N=40** | | | **Traditional learning sessions N=40** | | |
| --- | --- | --- | --- | --- | --- | --- |
|  | Completed assessments | Dropout | *p* | Completed assessments | Dropout | *p* |
| Gender (% women) | 58% | 63% | 1.00 | 63% | 47% | 0.46 |
| Examination grade in 2^nd^ year respiratory basics module | 12.25±2.44 | 13.39±2.13 | 0.206 | 14.38±1.89 | 13.38±2.00 | 0.201 |

**Supplementary Table 2. Student feedback about test ordering and interpretation in traditional learning sessions**

|  | **Vertically integrated curriculum** | | | | | |
| --- | --- | --- | --- | --- | --- | --- |
|  | 3rd year  n=40 | | 4th year  n=56 | 5th year  n=78 | 6th year  n=32 | |
| 1. I need courses or training sessions on diagnostic test ordering and interpretation (y/n) | 92.5%/7.5% | 98.2%/1.8% | | 95.9%/5.1% | | 93.8%/6.3% |
| 2. I have already had the possibility to suggest a diagnosis test (*never: 1; rarely: 2, sometimes: 3; regularly: 4; systematically: 5*):   - In clinical examination reports - At bedside with an resident/senior - In clinical case presentations - During classroom instruction - During medical staff meetings | 1.50±0.75  2.00±1.26  1.26±0.59  1.53±0.82  3.05±0.90 | 1.84±0.93  2.36±1.27  1.52±0.79  2.05±0.97  2.80±1.13 | | 2.53±0.88  2.71±1.28  1.46±0.68  2.40±1.04  3.03±1.21 | | 3.09±0.89  3.28±1.37  2.16±1.17  2.84±0.92  2.84±0.95 |
